# UPLC-ESI-MS/MS Analysis and Evaluation of Antioxidant Activity of Total Flavonoid Extract from *Paeonia lactiflora* Seed Peel and Optimization by Response Surface Methodology (RSM)

**DOI:** 10.1155/2021/7304107

**Published:** 2021-02-18

**Authors:** Guiqin Shi, Jiaxin Shen, Tao Wei, Fei Ren, Famou Guo, Yuan Zhou

**Affiliations:** Zhengzhou University of Light Industry, Zhengzhou 450002, China

## Abstract

In this study, the ultrasound-assisted extraction (UAE) of flavonoid from *Paeonia lactiflora* seed peel was optimized by response surface methodology (RSM). Single-factor experiments and a three-factor three-level Box-Behnken design (BBD) were performed to explore the effects of the following parameters on flavonoid extraction: ethanol concentration (*X*_1_), liquid-solid ratio (*X*_2_), and ultrasonic time (*X*_3_). The results showed that the optimal flavonoid yield (10.9045 mg RE/g) was as follows: ethanol concentration 62.93%, ultrasonic time 64.56 min, and liquid-solid ratio 24.86 mL/g. The optimized extract of *P. lactiflora* seed shell was further analyzed by UPLC-ESI-MS/MS, and 20 main flavonoids were identified and quantified, among which protocatechuic acid, vanillic acid, 4-hydroxybenzoic acid, and 3,4-dihydroxybenzaldehyde had the highest content. Furthermore, the results of the antioxidant test showed that the *P. lactiflora* seed peel extract obtained under optimized UAE conditions exhibited good antioxidant activity. The experimental results showed that ultrasound-assisted extraction was a fast, efficient, and simple method for extracting active ingredients from *P. lactiflora* seed peel, thereby making this byproduct a promising source of compounds in food and healthcare sectors.

## 1. Introduction

Herbaceous peony (*Paeonia lactiflora* Pall.), which was a perennial root flower belonging to the Paeoniaceae family [1], cultivated as a traditional medicinal and ornamental plant in China with high ornamental value [2] for more than 3,900 years [3]. The two traditional Chinese medicines, called “Chishao” and “Baishao,” were both derived from the dry roots of herbaceous peony [4]. These two medicines have the functions of clearing heat, cooling blood, promoting blood circulation, removing blood stasis [5], and nourishing blood for regulating menstruation [6], respectively. *P. lactiflora* seeds were the main by-product of *P. lactiflora*, and almost all the *P. lactiflora* seeds had been just taken as industrial waste. During the newly past decade, the health and nutritional value of *P. lactiflora* seeds had received considerable attention in addition to their importance as a pharmacologically active functional ingredient [7, 8]. *P. lactiflora* seed peel was solid residue caused by the sowing and oil extraction process, which was not deeply developed and utilized, resulting in a great waste of resources. It had been previously reported that the *P. lactiflora* seed peel contain a high number of polyphenolic compounds, mainly oligomeric stilbenes [9, 10], which have good bioactivity and were important for *P. lactiflora* seed peel in potential industrial applications of functional foods. At present, there are few studies reported on *P. lactiflora* seed peel.

Early studies had shown that *P. lactiflora* contains a variety of flavonoids and glycosides [11], which played important roles in various ecological and physiological processes in plants, including pigmentation, UV absorption, antioxidation, defence responses, and signal transduction [12]. Therefore, it is increasingly popular to find more resources of *P. lactiflora* which can extract effective active ingredients and recycle waste such as *P. lactiflora* seed peel. Moreover, there were few reports on the recovery and utilization of *P. lactiflora* seed peel and the extraction of its flavonoids. Traditional extraction methods of flavonoids, including heating, boiling, or refluxing were time-consuming, costly, and inefficient. Therefore, it is necessary to develop a more efficient, cheap, and simple method for the extraction of flavonoids. Studies have shown that there are obvious differences between ultrasonic-assisted extraction and heated water extraction in morphological characteristics and optical rotation of chemical structure [13]. And ultrasonic extraction can maximize the biological functions of these compounds and improve the yield of active ingredients [14]. In addition, some studies have shown that ultrasonic-assisted extraction can effectively improve the yield of flavonoids, antioxidant capacity, and scavenging capacity of reactive oxygen species [15–17]. In recent years, ultrasonic-assisted extraction has been gradually applied to the extraction of a variety of natural bioactive substances, especially flavonoids, because of its high extraction efficiency.

In the present study, ultrasound-assisted extraction for the flavonoid-enriched extract from *P. lactiflora* seed peel was investigated and the operational parameters (ethanol concentration, liquid-solid ratio, and ultrasonic time) were optimized using RSM with three-factor and three-level Box-Behnken design. Particularly, the extracts were also analyzed by UPLC-ESI-MS/MS to identify and quantify major flavonoid profiles. Furthermore, the antioxidant activity of flavonoids from *P. lactiflora* seed peel was also determined in vitro. The purpose of this study was to establish the optimal ultrasonic-assisted extraction conditions of flavonoids from *P. lactiflora* and provide extraction conditions and biological activity information for the development and utilization of flavonoids from *P. lactiflora*.

## 2. Material and Methods

### 2.1. Chemicals

Acetonitrile, methyl alcohol, and formic acid were HPLC grade and purchased from Merck (TGSHIELD, China). Standards were purchased from Shanghai Yuanye Biotechnology Co., Ltd. (Shanghai, China). ABTS, DPPH, neocuproine, and trolox were purchased from Shanghai Aladdin Bio-Chem Technology Co., Ltd. (Shanghai, China). All chemicals used in this study were analytical grade.

### 2.2. Plant Material

The variety of *Paeonia lactiflora* is *Youshao No.1*, which is a new variety bred by cross between *Chishao Paeonia lactiflora* and *Hang Paeonia lactiflora*. It was identified as *P. lactiflora* by Professor Yawei Han of Zhengzhou University of light industry. *Paeonia lactiflora* seed peel obtained from *P. lactiflora* planting base, Yucheng county, Henan province, which was dried under natural ventilation until reaching constant weight, then milled into powders, passed through 200 mesh sieve and stored in an air-tight container for further use.

### 2.3. Optimization of UAE from P. lactiflora

#### 2.3.1. Single-Factor Experiments

To select the optimum ethanol concentration, different ethanol concentrations (50, 60, 70, 80, and 90%) were tested under the conditions of ultrasonic time 60 min, and liquid-solid ratio 25 mL/g. To choose the best liquid-solid ratio, different liquid-solid ratios (10, 15, 20, 25, and 30 mL/g) were studied under the following conditions: ethanol concentration 60% and ultrasonic time 60 min. Finally, the effect of ultrasonic time of 30, 40, 50, 60, and 70 min on the extraction yield was evaluated under the following conditions: ethanol concentration 60% and liquid-solid ratio 25 mL/g. When doing the single-factor experiment, we found that there was no way to control the temperature in the actual operation. The actual temperature of the ultrasonic was higher than the temperature set by the instrument, and the temperature was very high, and the temperature changes greatly. The set temperature was the same every time, but the actual temperature is different. So we connected a constant temperature water bath to keep the water circulating, so that the water temperature was always controlled at 50°C and the ultrasonic power was 350 W. Therefore, the effect of ultrasonic temperature and power on flavonoid extraction was not considered.

#### 2.3.2. RSM Design

Based on the single-factor experimental experiments, a three-factor and three-level Box-Behnken design (BBD) with response surface methodology (RSM) was employed in this study to determine the optimal combination of the independent variables. The BBD procedure of RSM resulted in a total of 17 randomized experiments, and a quadratic model used to analyze experimental data was shown as follows:

(1) $$\begin{array}{c} Y=\beta_{0}+\overset{3}{\underset{i=1}{\sum }}\beta_{i}\;X_{i}+\overset{3}{\underset{i=1}{\sum }}\beta_{ii}X_{i}^{2}+\overset{2}{\underset{i=1}{\sum }}\overset{3}{\underset{j=i+1}{\sum }}\beta_{ij}X_{i}X_{j}+\varepsilon , \end{array}$$

where *Y* represents the predicted response, *X*_*i*_ and *X*_*j*_ are the level of variables, *β*_0_, *β*_*i*_, *β*_*ii*_, and *β*_*ij*_ are the intercept, linear, quadratic, and interactive terms, respectively, and *ε* is the error.

### 2.4. Measurement of Total Flavonoid Content (TFC)

According to the report of Aktumsek et al. [18], the content of total flavonoids in the extract was determined by spectrophotometry. Rutin was used as a standard, and the content of total flavonoids in the extract was expressed as milligram rutin equivalent per gram of extract (mg RE/g of sample).

### 2.5. UPLC-ESI-MS/MS Analysis

Chromatographic conditions: the UPLC analysis was conducted using a chromatograph (AB SCIEX) equipped with a binary pump, autosampler, column heater, solvent delivery system, diode array detector (DAD-270 nm), and data processing system. Waters UPLC HSS T3 (100 mm × 2.1 mm) with a particle size of 1.8 *μ*m was used for the analysis of flavonoids. Flavonoids were separated using a gradient elution with a mobile phase of 0.1% formic acid in ultrapure water (A) and acetonitrile (B). The gradient program was as follows: 0-2 min, 100%A; 2-30 min, 100%A-50%A; 30-32 min, 50%A-5%A; 32-34 min, 5%A; 34-34.1 min, 5%A-100%A; 34.1-35.5 min, 100%A. The flow rate was 0.3 mL/min, injected volume was 5 *μ*L, run time was 35.5 min, column temperature was 40°C, and wavelength used for monitoring was 270 nm.

Mass spectrometry conditions: the AB SCIEX QTRAP 6500+ mass spectrometry system was used to analyze the multiple reaction detection (MRM) mode under positive and negative ion scanning using an electrospray ion source (ESI), which greatly improves sensitivity. The electrospray ion source is positive and negative double ion source mode: positive ion mode: CUR: 35; EP: 10; IS:5500; CXP: 10; TEM:600°C; Gas1:60; Gas2:50; anion mode: CUR:35; EP:-10; IS:-4500; CXP:-20; TEM:600°C; Gas1:60; Gas2:50.

The chromatographic analysis of each replicate sample was performed in triplicate, and the average peak areas were used in calculations. Standards and samples were dissolved with UPLC grade methanol, and the solution at a concentration configured.

### 2.6. Evaluation of Antioxidant Activity

#### 2.6.1. Assay of Total Antioxidant Capacity

Assess according to the method of Prieto et al. [19]. Trolox was used as a positive control in this work. The percentage of total antioxidant capacity was calculated using the following equation:

(2) $$\begin{array}{c} \text{Antioxidant activity }\left(\%\right)=\frac{A_{X}-A_{0}}{A_{S}-A_{0}}\times 100, \end{array}$$

where *A*_0_ represents the absorbance of the reaction system without the extract, *A*_*s*_ represents the absorbance of ascorbic acid, and the *A*_*X*_ represents the absorbance of the reaction system in the presence of the extract.

#### 2.6.2. Assay of DPPH^·^ Radical Scavenging Activity

Assess according to the method of Yen and Chen [20]. Trolox was used as a positive control in this work. The percentage of DPPH^·^ radical scavenging was calculated using the following equation:

(3) $$\begin{array}{c} \text{Radical scavenging activity }\left(\%\right)=\frac{A_{0}-A_{X}}{A_{0}}\times 100, \end{array}$$

where *A*_0_ represents the absorbance of the reaction system without the extract and *A*_*X*_ represents the absorbance of the reaction system in the presence of the extract.

#### 2.6.3. Assay of ABTS+^·^ Radical Scavenging Activity

Assess according to the method of Re et al. [21]. Trolox was used as a positive control in this work, and the percentage of ABTS+^·^ radical scavenging was calculated using Equation (3).

#### 2.6.4. Assay of Cu^2+^ Reduction Ability

Assess according to the method of Apak et al. [22]. Trolox was used as a positive control in this work. The percentage of Cu^2+^ reduction ability was calculated using the following equation:

(4) $$\begin{array}{c} \text{Reduction ability }\left(\%\right)=\frac{A-A_{0}}{A_{\max}-A_{0}}\times 100, \end{array}$$

where *A*_0_ represents the absorbance of the reaction system without the extract, *A*_max_ represents the maximum absorbance of the reaction system in the presence of the extract, and *A* represents the absorbance of the reaction system in the presence of the extract.

### 2.7. Statistical Analysis

The Design-Expert® software version 10 (Stat-Ease, Inc., Minneapolis, MN, USA) was used to perform the RSM design and statistical analysis.

## 3. Results and Discussion

### 3.1. Single-Factor Experiments

#### 3.1.1. Effect of Ethanol Concentration on the TFC

Overall, ethanol concentration was one of the influential factors affecting the total flavonoid content and was an important indicator to evaluate the extraction efficiency. To investigate the effect of ethanol concentration on the extraction of flavonoids, a solvent concentration range of 50-90% was tested in the present experiment.

**Figure 1:**
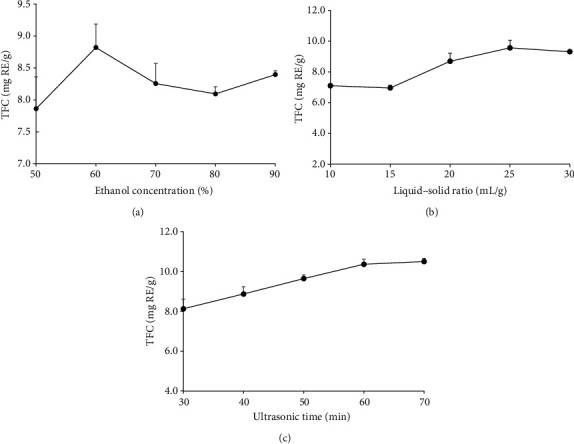
Effects of three independent variables on the total flavonoid content (TFC): (a) ethanol concentration; (b) liquid-solid ratio; (c) ultrasonic time.

As shown in Figure 1(a), the TFC increased dramatically when the ethanol concentration varied from 50% to 60%. The TFC showed a downward trend when the ethanol concentration was 60% to 80%, but the TFC increased slightly when the ethanol concentration was 80% to 90%. Overall, TFC reached its maximum (8.82 ± 0.37 mg RE/g) at an ethanol concentration of 60%, which was possibly due to the fact that higher ethanol concentrations are more likely to lead to the dissolution of alcohol-soluble impurities, thus affecting the dissolution of flavonoids [23]. Therefore, 50–70% ethanol solution was selected for further BBD experiments.

#### 3.1.2. Effect of the Liquid-Solid Ratio on the TFC

The effect of the liquid-solid ratio (10-30 mL/g) on the TFC is shown in Figure 1(b). TFC increased remarkably when the liquid-solid ratio varied from 15 to 25 mL/g and reached a maximum (9.56 ± 0.50 mg RE/g) at 25 mL/g. However, the liquid-solid ratio exhibited a slow downward trend when the liquid-solid ratio was above 25 mL/g, which was possible that the contact area was saturated with increasing ethanol volume. Similar results on the effect of the solid-liquid ratio on the extraction of phenolic compounds from grape pomace were also reported by Pinelo et al. [24]. Therefore, the liquid-solid ratio range of 20–30 mL/g was chosen for further BBD experiments.

#### 3.1.3. Effect of Ultrasonic Time on the TFC

Ultrasonic time was another important factor that can remarkably influence TFC. In this study, the influence of ultrasonic time (30-70 min) on the TFC was researched. As shown in Figure 1(c), the TFC increased with the increasing ultrasonic time and reached a maximum (10.37 ± 0.26 mg RE/g) at 60 min. When the ultrasonic time continued to rise to more than 60 minutes, TFC content increased slowly. Moreover, when the ultrasonic time exceeds 60 minutes, the yield increases slowly, while the long ultrasonic time will increase the extraction cost. Hence, the ultrasonic time within 50–70 min was selected for subsequent BBD experiments.

### 3.2. Optimization of UAE Process by RSM

#### 3.2.1. Model Fitting and Statistical Analysis

The three-level and three-factor BBD was carried out to optimize the UAE of flavonoids from *P. lactiflora* seed peel. BBD matrix and response values for the flavonoid yield are listed in Table 1. The results of the ANOVA are shown in Table 2. According to the existing experimental results, statistical regression analysis using Design-Expert software resulted in the following second-order polynomial equation:

(5) $$\begin{array}{c} Y=11.06+0.21X_{1}+0.33X_{2}+0.011X_{3}-0.13X_{1}X_{2}+0.14X_{1}X_{3}-0.048X_{2}X_{3}-0.55X_{1}^{2}-0.82X_{2}^{2}-0.89X_{3}^{2}, \end{array}$$

where *X*_1_, *X*_2_, and *X*_3_ are the ethanol concentration, liquid-solid ratio, and ultrasonic time, respectively. *Y* is the predicted value of TFC.

The values of linear coefficients of the second-order polynomial equation for *X*_1_, *X*_2_, and *X*_3_, as well as of the interaction coefficients *X*_1_*X*_3_, were all positive, suggesting that the increase of these parameters results in a favorable effect on the extraction of TFC. However, the negative values of the interaction coefficients *X*_1_*X*_2_ and *X*_2_*X*_3_, as well as of the quadratic coefficients (*X*_1_^2^, *X*_2_^2^, and *X*_3_^2^, respectively), imply that reached a maximum value before decreasing for high values of those three extraction parameters. The *F* value of the model of 82.17 implies the model was very significant (*p* < 0.001). There was only a 0.01% chance that a model's *F* value this large could occur due to noise. Values of “Prob > *F*” less than 0.0500 indicate that model terms were significant. In this case, *X*_1_, *X*_2_, *X*_3_, *X*_1_*X*_3_, *X*_1_^2^, *X*_2_^2^, and *X*_3_^2^ were significant model terms.

The lack of fit of each model was not significant (*p* > 0.05), indicating that the developed model adequately explains the relationship between the independent variables and responses. The lack of fit *F* value of 2.96 implies that the lack of fit was not significant relative to the pure error. There was a 16.11% chance that a lack of fit *F* value this large could occur due to noise. The predicted *R*^2^ of 0.8920 was in reasonable agreement with the adjusted *R*^2^ of 0.9786, implying that the predicted values were highly consistent with the experimental values. Adequate precision measured the signal-to-noise ratio, in which a ratio greater than 4 was desirable. The ratio of 23.971 indicated an adequate signal. This model could be used to navigate the design space.

#### 3.2.2. Response Surface Analysis

The three-dimensional (3D) response surface images illustrate the mutual influence of any two independent variables on the dependent variable, and the shape of the 3D response surface plots provides information on the influence degree [25].

**Figure 2:**
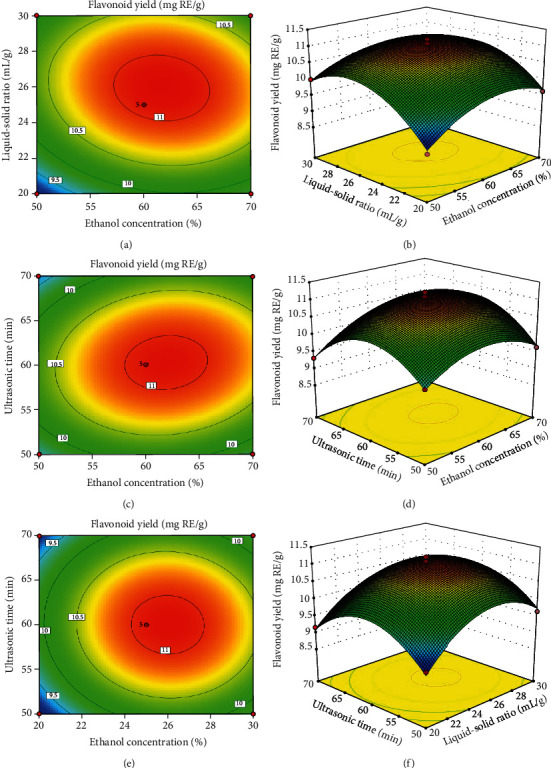
Response surface plots (a, c, e) and contour plots (b, d, f) of flavonoid yield affected by extraction temperature (*X*_1_), liquid-solid ratio (*X*_2_), and ultrasonic time (*X*_3_).

Figure 2 offers a visual interpretation of the interactions between two variables (*X*_1_*X*_2_, *X*_1_*X*_3_, and *X*_2_*X*_3_) on the response variable (*Y*). The interaction between *X*_1_ and *X*_2_, *X*_1_ and *X*_3_, and *X*_2_ and *X*_3_ was relatively significant, respectively. Figures 2(a) and 2(b) show the influences of ethanol concentration (*X*_1_) with the liquid-solid ratio (*X*_2_) on the extraction yields of flavonoid yield. The initial increase of ethanol concentration (50% to about 60%) led to an increase in TFC and was followed by a decline thereafter (about 60% to 70%). Similarly, a rapid rise in TFC was obtained when the liquid-solid ratio varied from 20 to about 25 mL/g; then, the extraction yield of TFC was decreased slowly with the increasing liquid-solid ratio. Figures 2(c) and 2(d) show the influences of ethanol concentration (*X*_1_) with ultrasonic time (*X*_3_) on the extraction yields of flavonoid yield. The initial increase of ethanol concentration (50% to about 60%) led to an increase in TFC and followed by a decline thereafter (about 60% to 70%). Similarly, a rapid rise in TFC was obtained when ultrasonic time varied from 50 to about 60 min; then, the extraction yield of TFC was decreased slowly with increasing ultrasonic time. Figures 2(e) and 2(f) show the influences of the liquid-solid ratio (*X*_2_) with ultrasonic time (*X*_3_) on the extraction yields of flavonoid yield. The initial increase of the liquid-solid ratio (20 to about 25 mL/g) led to an increase in TFC and was followed by a decline thereafter (about 25 to 30 mL/g). Similarly, a rapid rise in TFC was obtained when ultrasonic time varied from 50 to about 60 min; then, the extraction yield of TFC was decreased slowly with increasing ultrasonic time. The analysis of the results showed that the optimal process conditions for the extraction of total flavonoids from *P. lactiflora* seed peel were within the designed experimental range. This was basically consistent with the results found by Ghafoor et al. [26].

Optimization of ethanol concentration, liquid-solid ratio, and ultrasonic time was important for the extraction of flavonoids from *P. lactiflora* seed peel. Ethanol was preferred as a solvent in the food industry and was regarded as a dietary alcohol; however, excess ethanol can lead to denaturation of flavonoids. Ultrasound offered a mechanical effect allowing greater penetration of solvent into the sample matrix, increasing the contact surface area between the solid and liquid phase, and as a result, the solute quickly diffuses from the solid phase to the solvent. However, an excessive liquid-solid ratio can hinder the dissolution of flavonoids. Extraction time was an important parameter in ultrasound-assisted extraction. It had been found that prolonged extraction time may lead to increased degradation of bioactive compounds in the case of ultrasound-assisted extraction [27]. During the extended extraction time, especially in the presence of water, it had been shown to induce the oxidation of polyphenols, thus significantly reducing the antioxidant capacity of the resulting extracts [28]. Also, shortening the extraction time to reduce energy consumption is of great concern at present.

#### 3.2.3. Validation of the Optimized Model

The optimal conditions for flavonoid extraction were as follows: ethanol concentration of 62.93%, liquid-solid ratio of 24.86 mL/g, and ultrasonic time of 64.56 min. Under these conditions, the predicted value was 10.9045 mg/g. Subsequently, in order to verify the reliability of the predictive model, validation experiments were conducted under optimized extraction conditions. The obtained experimental values were 10.37 ± 0.34 mg/g, which confirmed that the model was accurate and reliable.

### 3.3. Analysis of Flavonoids in the Extracts by UPLC-ESI-MS/MS

**Figure 3:**
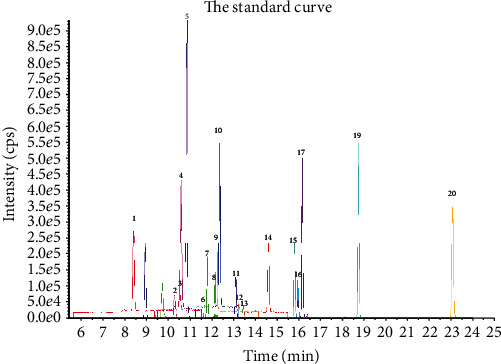
UPLC-ESI-MS/MS profiles of standard mixture.

**Figure 4:**
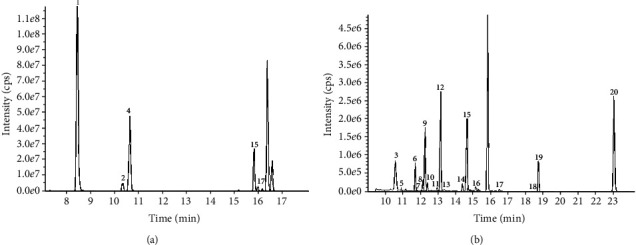
*Paeonia lactiflora* seed peel extract obtained by optimized ultrasonic-assisted extraction. Peak identification: 1: protocatechuic acid; 2: 3,4-dihydroxybenzaldehyde; 3: gentisic acid; 4: 4-hydroxybenzoic acid; 5: aesculin; 6: catechin; 7: chlorogenic acid; 8: cryptochlorogenic acid; 9: vanillic acid; 10: caffeic acid; 11: syringic acid; 12: aesculetin; 13: epicatechin; 14: 4-hydroxycinnamic acid; 15: transpiceid; 16: rutin; 17: delphinidin 3-glucoside; 18: hesperidin; 19: phlorizin; 20: naringenin. (a) The peak with higher content in the extract. (b) The peaks of other flavonoids in the extract.

The flavonoids in ultrasonic-assisted extraction of *P. lactiflora* seed peel were analyzed by UPLC-ESI-MS/MS. The chromatogram of flavonoids identified is shown in Figures 3 and 4. The types and quantitation of these compounds are listed in Table 3. The results showed that a total of 20 flavonoids were detected in the extract of *P. lactiflora* seed peel. These data confirmed that there were abundant flavonoids in *P. lactiflora* seed peel.

The results of quantitative analysis showed that the content of flavonoids in *P. lactiflora* seed peel extract was protocatechuic acid (186217.33 ng/g), vanillic acid (51672.94 ng/g), 4-hydroxybenzoic acid (50510.04 ng/g), and 3,4-dihydroxybenzaldehyde (32983.74 ng/g), respectively. The precursor ion spectra at *m*/*z* 152.9 and the product ion spectra at *m*/*z* 108.9 (RT = 8.42; peak no. 1), based on reference standards, this flavone was identified as protocatechuic acid. The precursor ion spectra at *m*/*z* 353.2 and the product ion spectra at *m*/*z* 190.9 (RT = 12.22; peak no. 9), this flavone was identified as vanillic acid. The precursor ion spectra at *m*/*z* 177 and the product ion spectra at *m*/*z* 132.9 (RT = 14.59; peak no. 14), this flavone was identified as 4-hydroxybenzoic acid. The precursor ion spectra at *m*/*z* 307 and the product ion spectra at *m*/*z* 139.3 (RT = 10.3; peak no. 2), this flavone was identified as 3,4-dihydroxybenzaldehyde. The identification information of other components is shown in Table 3.

There was no doubt that the pharmacological action of *P. lactiflora* seed peel depends on its chemical composition. The specific pharmacological activity of these compounds needs to be further validated. Li et al. detected 26 flavonoids in *Paeonia Section Moutan* [29], which is similar to the amount of flavonoids we detected. These results indicated that Paeoniaceae plants may contain rich flavonoids and other active components, which will lay a theoretical foundation for the development and utilization of Paeoniaceae plants.

### 3.4. Antioxidant Activity

**Figure 5:**
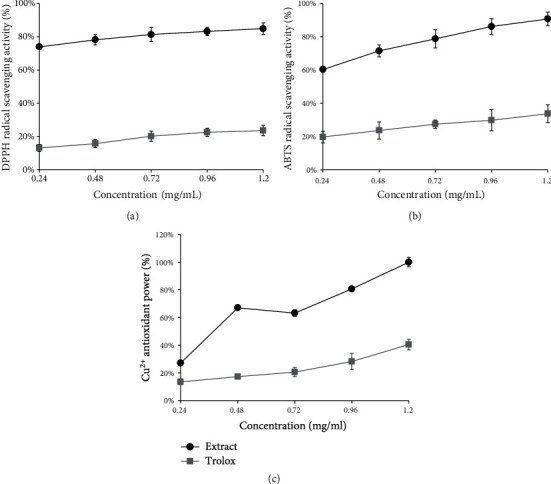
Antioxidant capacity of *Paeonia lactiflora* seed peel extract obtained by optimized ultrasound-assisted extraction compared with trolox: (a) DPPH^·^ radical scavenging activity; (b) ABTS^+·^ radical scavenging activity; (c) Cu^2+^ reduction ability.

**Figure 6:**
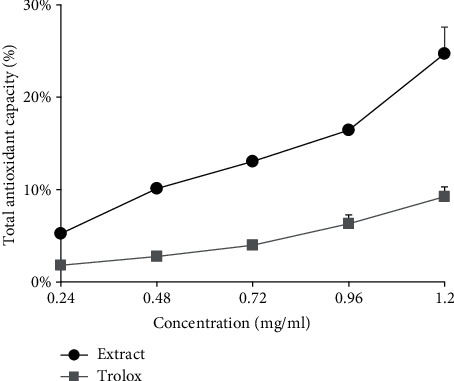
Total antioxidant capacity of *Paeonia lactiflora* seed peel extract obtained by optimized ultrasound-assisted extraction compared with trolox.

The antioxidant activity of flavonoids could be evaluated by different methods of scavenging free radicals or delaying the generation of free radicals in vitro, including the determination of the DPPH^·^ radical scavenging activity, ABTS+^·^ radical scavenging activity, Cu^2+^ reduction ability assay (CUPRAC), and total antioxidant capacity. The antioxidant potential of *P. lactiflora* seed peel extracts in terms of its free radical scavenging (DPPH and ABTS), reducing power (CUPRAC), is presented in Figure 5, and total antioxidant capacity is presented in Figure 6.

It was well known that flavonoids possess many biochemical properties, but the best-described property of almost every group of flavonoids is their capacity to act as antioxidants [30]. A comparison of the trolox and the *P. lactiflora* seed peel extract obtained by optimized UAE was performed, and the results of the total antioxidant capacity assay are shown in Figure 4. It was observed that the total antioxidant effect of the *P. lactiflora* seed peel extract was increased with increasing concentration, which is in a dose-dependent manner. The *P. lactiflora* seed peel extract at the test concentrations of 0.24, 0.48, 0.72, 0.96, and 1.2 mg/mL showed 5.21 ± 0.35%, 10.12 ± 0.19%, 13.05 ± 0.28%, 16.43 ± 0.06%, and 24.70 ± 2.88% total antioxidant capacity, respectively. It had been shown that antioxidant capacity was strongly positively correlated with total flavonoid content [31]. Quercetin have 5,7- and 3′,4′-dihydroxyl groups in the A and B ring, respectively, and the 3-hydroxyl group of flavonol enhances the antioxidant activity [32]. Therefore, among common aglycones, the antioxidant activity decreases in the order quercetin > kaempferol > luteolin > apigenin, and the antioxidant activity of their glycosides decreases slightly [33]. In this study, quercetin was the second main compound and the most abundant for the *P. lactiflora* seed peel; therefore, it might be also considered as the main compound that contributes to the high antioxidant capacity observed in all of the studied extracts.

A comparison of the trolox and the *P. lactiflora* seed peel extract obtained by optimized UAE was performed, and the results of the DPPH^·^ radical scavenging activity assay are shown in Figure 5(a). It was observed that the DPPH^·^ radical scavenging effect of the *P. lactiflora* seed peel extract was basically unchanged with increasing concentration. The *P. lactiflora* seed peel extract at the test concentrations of 0.24, 0.48, 0.72, 0.96, and 1.2 mg/mL showed 78.89 ± 1.13%, 84.50 ± 3.15%, 83.32 ± 4.24%, 83.18 ± 2.45%, and 84.84 ± 3.46% DPPH^·^ radical scavenging, respectively. It was known that only flavonoids of a certain structure and particularly hydroxyl position in the molecule determine antioxidant properties [34]. The basically unchanged radical scavenging activity of DPPH may be attributed to some specific flavonoids. The results indicated that the UAE extract of *P. lactiflora* seed peel had a good potential for scavenging DPPH^·^ radical.

A comparison of the trolox and the *P. lactiflora* seed peel extract obtained by optimized UAE was performed, and the results of the ABTS+^·^ radical scavenging activity assay are shown in Figure 5(b). It was found that the ABTS+^·^ radical scavenging effect of the *P. lactiflora* seed peel extract was increased with increasing concentration, which is in a dose-dependent manner. The *P. lactiflora* seed peel extract at the test concentrations of 0.24, 0.48, 0.72, 0.96, and 1.2 mg/mL showed 60.31 ± 1.56%, 71.50 ± 3.60%, 78.74 ± 5.43%, 86.14 ± 4.81%, and 90.71 ± 4.17% ABTS+^·^ radical scavenging, respectively. It had been confirmed that flavonoids are likely to contribute to the radical scavenging activity of plant extracts [35]. From these results, it could be stated that the *P. lactiflora* seed peel extract was a good ABTS+^·^ radical scavenger.

The reduction capacity of a compound might serve as a significant indicator of its potential antioxidant activity. A comparison of the trolox and the *P. lactiflora* seed peel extract obtained by optimized UAE was performed, and the results of the Cu^2+^ reduction ability assay are shown in Figure 5(c). The reducing capacity of Cu^2+^ showed a trend of increasing first, then gradually decreasing, and finally increasing rapidly. The *P. lactiflora* seed peel extract at the test concentrations of 0.24, 0.48, 0.72, 0.96, and 1.2 mg/mL showed 27.14 ± 0.65%, 68.37 ± 2.68%, 63.21 ± 2.39%, 80.65 ± 1.62%, and 99.99 ± 3.42% Cu^2+^ reducing, respectively. Results indicated that *P. lactiflora* seed peel might serve as an excellent Cu^2+^ reducer.

It was known that differences in antioxidant activity in plant materials depend on the cultivar, growing environment, extraction method, and so on [36]. Li et al. studied 93 traditional Chinese herbal medicines and found that the highest DPPH scavenging activities of *P. lactiflora* were 94.48% [37]. Zhang et al. enriched the total flavonoids of *Paeonia ostii* flowers via polyamide resin columns and found that the extracts with higher total flavonoid content had higher antioxidant activity [38]. Fan et al. studied the flowers of 48 Zhongyuan tree peony and found that antioxidant activity varied among the cultivars and highly corresponded with total phenolic content [39]. Studies had shown significant differences in the ability of different organs of peony to scavenge free radicals, suggesting that there were significant differences in their antioxidant content [40]. In addition, a significant correlation was observed between individual flavonoid content and antioxidant activity. Among them, quercetin was negatively correlated with the antioxidant activity and luteolin had a significant positive correlation with the antioxidant activity [29, 41]. These results indicated that even flavonoids with known antioxidant properties show significantly different antioxidant activities when tested as part of a complex biological mixture.

Overall, the total antioxidant capacity, ABTS, DPPH, and FRAP experiments proved that the extracts have high antioxidant activity, which may be related to TPC. In addition, the findings demonstrated that the combination of two antioxidants may have strong antioxidant activity, but not necessarily engender synergistic efficacy; it may even generate antagonistic interaction [42]. Whether antagonistic or synergistic interactions occurred between the components in this study requires subsequent continued research. The results also had shown that the *P. lactiflora* seed peel could be used as a cheap and abundant source of antioxidants in the pharmaceutical and food industries.

## 4. Conclusions

In this study, single-factor experiments were carried out to determine the optimal extraction parameters. A three-level and three-factor BBD was performed to explore the quadratic effects by using RSM of the following parameters on flavonoids, and the process parameters optimized by RSM were as follows: ethanol concentration of 62.93%, liquid-solid ratio of 24.86 mL/g, and ultrasonic time of 64.56 min. Under these conditions, the predicted value was 10.9045 mg/g. Moreover, UPLC-ESI-MS/MS analysis showed that the main flavonoids contained in the *P. lactiflora* seed peel were protocatechuic acid, vanillic acid, 4-hydroxybenzoic acid, and 3,4-dihydroxybenzaldehyde. In addition, the *P. lactiflora* seed peel extract obtained through the optimized UAE method exhibited good antioxidant capacity. These results provided a theoretical basis for the comprehensive utilization of *Paeonia lactiflora* seed peel and the extraction of its flavonoids as a potential source of antioxidants.

## Figures and Tables

**Table 1:** Box-Behnken design and response values for the flavonoid yield of *P. lactiflora* seed peel.

Run	*X*_1_: ethanol concentration	*X*_2_: liquid-solid ratio	*X*_3_: ultrasonic time	*Y*: flavonoid yield
	(%)	(mL/g)	(min)	(mg RE/g)
1	70 (1)	30 (1)	60 (0)	10.206
2	70 (1)	25 (0)	50 (-1)	9.642
3	60 (0)	25 (0)	60 (0)	11.202
4	50 (-1)	20 (-1)	60 (0)	8.919
5	70 (1)	25 (0)	70 (1)	9.956
6	60 (0)	20 (-1)	50 (-1)	9.053
7	70 (1)	20 (-1)	60 (0)	9.633
8	60 (0)	20 (-1)	70 (1)	9.156
9	60 (0)	25 (0)	60 (0)	10.984
10	60 (0)	30 (1)	70 (1)	9.548
11	50 (-1)	25 (0)	50(-1)	9.556
12	50 (-1)	25 (0)	70 (1)	9.316
13	60 (0)	25 (0)	60 (0)	11.038
14	60 (0)	30 (1)	50 (-1)	9.636
15	50 (-1)	30 (1)	60 (0)	10.002
16	60 (0)	25 (0)	60 (0)	11.068
17	60 (0)	25 (0)	60 (0)	11.016

**Table 2:** Analysis of variance for the response surface model for the flavonoid yield of *P. lactiflora* seed peel.

Source	Sum of squares	Df	Mean square	*F* value	*p* value Prob > *F*
Model	9.66	9	1.07	82.17	<0.0001
*X*_1_	0.34	1	0.34	25.87	0.0014
*X*_2_	0.87	1	0.87	66.25	<0.0001
*X*_3_	9.901 × 10^−4^	1	9.901 × 10^−4^	0.076	0.7910
*X*_1_ *X*_2_	0.065	1	0.065	4.98	0.0609
*X*_1_ *X*_3_	0.077	1	0.077	5.87	0.0458
*X*_2_ *X*_3_	9.120 × 10^−3^	1	9.120 × 10^−3^	0.70	0.4310
*X*_1_^2^	1.28	1	1.28	97.94	<0.0001
*X*_2_^2^	2.83	1	2.83	217.00	<0.0001
*X*_3_^2^	3.36	1	3.36	257.04	<0.0001
Residual	0.091	7	0.013		
Lack of fit	0.063	3	0.021	2.96	0.1611
Pure error	0.028	4	7.103 × 10^−3^		
Cor total	9.75	16			
*R* ^2^	0.9906				
Adj *R*^2^	0.9786				
Pred *R*^2^	0.8920				
Adeq precision	23.971				

*X*_1_: ethanol concentration; *X*_2_: liquid-solid ratio; *X*_3_: ultrasonic time.

**Table 3:** The content of flavonoids in the extract of *P. lactiflora* seed peel was determined by UPLC-ESI-MS/MS (ng/g of extract).

Peak	RT (min)	Identification	Ethanol extract	Q1 (*m*/*z*)	Q3 (*m*/*z*)	CE
1	8.42	Protocatechuic acid	186217.33	152.9	108.9	-20
2	10.3	3,4-Dihydroxybenzaldehyde	32983.74	307	139.3	20
3	10.54	Gentisic acid	5491.66	310.9	148.9	-15
4	10.59	4-Hydroxybenzoic acid	50510.04	137.1	107.9	-27
5	10.85	Aesculin	30.91	153	108.8	-18
6	11.66	Catechin	6294.43	136.9	93	-20
7	11.79	Chlorogenic acid	77.62	341.1	179.1	21
8	12.15	Cryptochlorogenic acid	30.18	288.9	109	20
9	12.22	Vanillic acid	51672.94	353.2	190.9	-20
10	12.36	Caffeic acid	145.70	353.1	172.9	-20
11	13.1	Syringic acid	303.37	166.9	107.9	-25
12	13.24	Aesculetin	1182.14	179	135	-25
13	13.44	Epicatechin	526.28	199	140.2	20
14	14.59	4-Hydroxycinnamic acid	4135.44	177	132.9	-25
15	15.79	Transpiceid	8195.52	288.8	109	-35
16	15.95	Rutin	63.34	163	119.1	-20
17	16.15	Delphinidin 3-glucoside	997.07	389.1	227.1	-25
18	18.11	Hesperidin	62.82	609.1	300.2	-46
19	18.71	Phlorizin	737.92	465.2	303.1	25
20	22.9	Naringenin	2920.60	609.2	325.3	-40

Q1: the precursor ion; Q3: the product ion.

## Data Availability

The data used to support the findings of this study are included in the manuscript.
